# Electrolyte and Acid-Base Disorders in the Renal Transplant Recipient

**DOI:** 10.3389/fmed.2018.00261

**Published:** 2018-10-02

**Authors:** Vaishnavi Pochineni, Helbert Rondon-Berrios

**Affiliations:** Department of Medicine, University of Pittsburgh, Pittsburgh, PA, United States

**Keywords:** kidney transplant, electrolytes, metabolic acidosis, hypophosphatemia, hypomagnesemia, hypercalcemia, hyperkalemia, renal transplant

## Abstract

Kidney transplantation is the current treatment of choice for patients with end-stage renal disease. Innovations in transplantation and immunosuppression regimens have greatly improved the renal allograft survival. Based on recently published data from the Scientific Registry of Transplant recipients, prevalence of kidney transplants is steadily rising in the United States. Over 210,000 kidney transplant recipients were alive with a functioning graft in mid-2016, which is nearly twice as many as in 2005. While successful renal transplantation corrects most of the electrolyte and mineral abnormalities seen in advanced renal failure, the abnormalities seen in the post-transplant period are surprisingly different from those seen in chronic kidney disease. Multiple factors contribute to the high prevalence of these abnormalities that include level of allograft function, use of immunosuppressive medications and metabolic changes in the post-transplant period. Electrolyte disturbances are common in patients after renal transplantation, and several studies have tried to determine the clinical significance of these disturbances. In this manuscript we review the key aspects of the most commonly found post-transplant electrolyte abnormalities. We focus on their epidemiology, pathophysiology, clinical manifestations, and available treatment approaches.

## Introduction

End-stage renal disease is associated with a variety of metabolic and electrolyte derangements. Although most of these abnormalities resolve with kidney transplantation, a new plethora of electrolytes and acid-base abnormalities are noted. With rising prevalence of kidney transplantation, even the general nephrologist and internists have to address these abnormalities. An important study conducted by Einollahi et al. shed some light about the most common electrolyte disorders encountered after kidney transplantation, but the exact incidence and prevalence of these disorders vary from study to study (1). The authors observed that the most frequent post-transplant electrolyte and acid-base disturbances are hyperkalemia, metabolic acidosis, hypercalcemia, hypomagnesemia, and hypophosphatemia. We will proceed to further describe the epidemiology, pathophysiology, clinical manifestations, and treatment options of these specific disorders based on the available clinical evidence.

## Hyperkalemia

### Epidemiology

Hyperkalemia is a common complication in renal allograft recipients found with a reported incidence ranging from 25 to 44% in kidney transplant recipients on calcineurin inhibitors[CNIs] (2, 3). Simultaneous kidney-pancreas transplant recipients with bladder drainage are reported to have more frequent hyperkalemia, with one study reporting an incidence of 73% (3). Studies are few that describe the time course of hyperkalemia after kidney transplantation. In a small study published in 1996 in type 1 diabetics, hyperkalemia occurred up to average post-operative day of 100 for kidney transplant recipients. Only two patients had hyperkalemia beyond 8 months after transplantation (3). Patients on tacrolimus have more frequent hyperkalemia when compared to patients on cyclosporine (4).

### Pathophysiology

**Figure 1:**
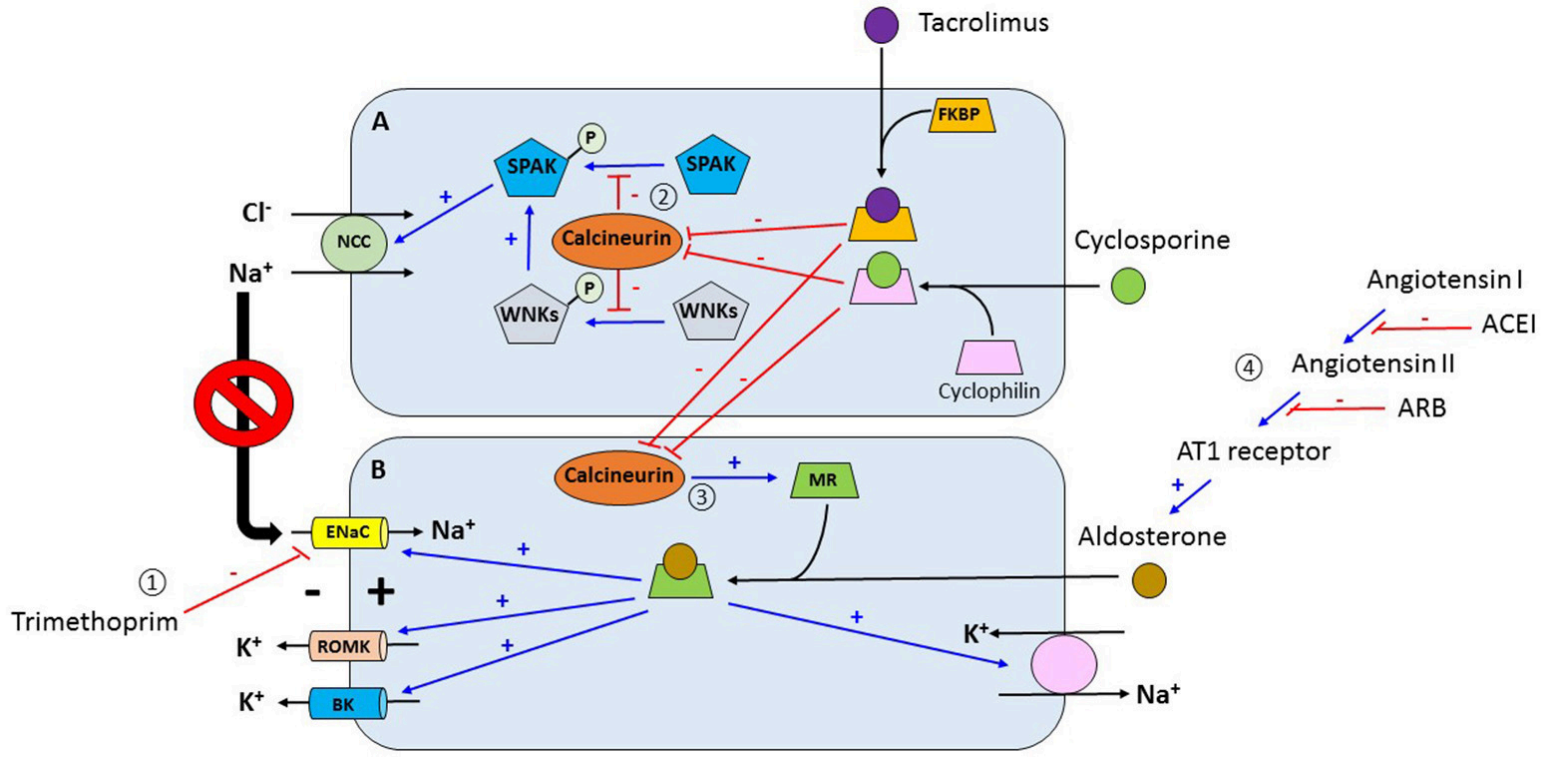
(1) Trimethoprim inhibits the activity of ENaC in the late distal convoluted convoluted tubule and cortical collecting duct which decreases the electrical gradient for channels. (2) Tacrolimus and cyclosporine bind to FKBP and cyclophilin respectively forming complexes. These complexes inhibit calcineurin which is a phosphatase. Under normal conditions, calcineurin retrieve phosphate groups from different proteins including SPAK and WNKs. Inhibition of calcineurin allows for the phosphorylation of these kinases which activate NCC increasing sodium chloride reabsorption in the distal convoluted tubule. Increasing sodium reabsorption in this nephron segment decreases the delivery of sodium to more distal segments which in turn decreases the electrical gradient for potassium secretion via ROMK channels. (3) Activation of MR by aldosterone increases the activity of proteins associated with potassium excretion in the distal nephron including ENaC, ROMK, BK, and the Na+-K+-ATPase pump. In addition, calcineurin increases the expression of the mineralocorticoid receptor. In contrast, tacrolimus and cyclosporine, by inhibiting calcineurin, decrease the decrease the expression of the mineralocorticoid receptor with subsequent reduction in potassium Angiotensin converting enzyme inhibitors and angiotensin receptor blockers inhibit aldosterone with the subsequent reduction in potassium excretion. ENaC, epithelial sodium channel; ROMK, renal outer medullary potassium channel; FKBP, FK binding protein; SPAK, STE20/SPS1-related proline- and alanine-rich kinase; WNKs, with no-lysine kinases; NCC, sodium chloride cotransporter; MR, mineralocorticoid receptor; BK, big potassium channel. **(A)** Distal convoluted tubular cell. **(B)** Principal cell of collecting duct.

Potassium (K+) is the most abundant intracellular cation with a concentration of 150 mEq/L compared to just 4 mEq/L in the extracellular compartment. This difference in concentration between compartments is maintained by the Na-K-ATPase pump. Since the ratio between the intracellular and extracellular K+ concentration is the main determinant of the resting membrane potential then maintaining K homeostasis, i.e., maintaining a relatively constant plasma K+ concentration, is critical for cell function (5). Plasma K+ concentration is determined by the relationship among K+ intake, its distribution between the intracellular and extracellular compartments, and renal K+ excretion. Under normal conditions, a dietary K+ load is absorbed by the gut into the circulation followed by a rapid uptake by muscle and liver cells facilitated by the presence of insulin and beta 2 adrenergic receptors after which the K+ load is mostly renally excreted, a process that is primarily determined by potassium secretion by the principal cells located in the connecting tubule and cortical collecting duct. K+ secretion in these segments is tightly regulated. Adequate aldosterone secretion and responsiveness and adequate distal water and sodium delivery are key determinants of renal K+ secretion (5). Distal sodium delivery is especially important because sodium reabsorption via the epithelial sodium channel (ENaC) in these distal nephron segments creates an electrical gradient that favors K+ secretion. Common mechanisms for hyperkalemia in the non-transplant individual are insulin deficiency, inorganic metabolic acidosis, decreased glomerular filtration rate, and decreased distal sodium delivery. Hyperkalemia in the kidney transplant recipient is usually seen in association with renal tubular acidosis and can be seen even without any of the above-mentioned factors. Insulinopenia or insulin resistance can decrease translocation of potassium and glucose from the extracellular to the intracellular compartment and cause hyperkalemia and hyperglycemia in the post-transplant setting, especially in insulin dependent diabetics (6). Figure 1 illustrates the most common mechanisms of hyperkalemia in the renal transplant recipient. Medications used post-transplant are thought to be the major cause for post-transplant hyperkalemia in recipients with a well-functioning graft. Use of trimethoprim in Trimethoprim/Sulfamethoxazole (TMP/SMX) in standard doses contributes to hyperkalemia by ENaC blockade (7) but the incidence is low especially when the regimen comprises of single strength tablet three times weekly for Pneumocystis and urinary tract infection prophylaxis (8). Use of pentamidine can also cause hyperkalemia by a similar mechanism (9). Use of renin-angiotensin system blockers is associated with better patient and graft survival in renal transplant recipients but risk of life threatening hyperkalemia is also 2-fold when compared to recipients not on these medications (10). Calcineurin inhibitors (CNIs) such as tacrolimus and cyclosporine are considered the major players in the development of hyperkalemia in the kidney transplant recipient. Deppe and Heering showed that CNIs inhibit mineralocorticoid receptor transcriptional activity, causing downregulation of mineralocorticoid expression leading to impaired mineralocorticoid function and aldosterone resistance (11, 12). Hence, patients on CNIs might show signs of hypoaldosteronism despite normal plasma aldosterone levels. Hoorn EJ et al. postulated a new mechanistic pathway of hyperkalemia in transplant recipients by demonstrating that tacrolimus activates the thiazide-sensitive sodium-chloride cotransporter (NCC) in the distal convoluted tubule (DCT) leading to hyperkalemia and hypertension similar to the ones that occur in Gordon syndrome (13). What is more, it seems that the tacrolimus causes this effect predominantly by directly inhibiting calcineurin in DCT cells (14). If this concept is further validated in future studies, thiazide diuretics may become an attractive and more targeted therapeutic option for hyperkalemia in these patients.

### Clinical manifestations

Hyperkalemia is usually asymptomatic until cardiac complications develop. Excitable tissues like cardiac and skeletal muscle are the most commonly affected organs. Hyperkalemia causes increased potassium conductance and shortens the duration of the action potential making the heart more susceptible to arrhythmias. Arrhythmias like sinus arrest, sinus bradycardia, ventricular fibrillation and asystole can be caused by hyperkalemia (15). Peaked T-waves and a prolonged PR segment are seen initially on the EKG, and in severe cases progress to widening of the QRS complex, a sine-wave appearance and asystole (15). Hyperkalemia can also rarely cause paralysis, myopathy and paresthesia. Transplant specific consequences of hyperkalemia have not yet been noted or outlined.

### Treatment

Limiting the use or lowering the dose of drugs that increase potassium levels may not always be feasible in the immediate post-transplant setting. Initial approach should include dietary modification and addition of thiazide or loop diuretic if volume status permits. Fludrocortisone has mineralocorticoid properties and causes sodium reabsorption and potassium excretion in the distal nephron. Studies indicate that fludrocortisone can ameliorate persistent hyperkalemia and metabolic acidosis in transplant recipients, but it can result in fluid retention (11, 16). Sodium polystyrene sulfonate is a cation exchange resin that has been commonly used to treat chronic hyperkalemia for decades. Its use is associated with colonic perforation, which is a rare yet catastrophic complication after renal transplantation, mainly when used with sorbitol as a carrier. Hence, caution is advised in its use, especially in the immediate peri-operative period (17, 18). Patiromer, a relatively new cation exchange resin which exchanges potassium for calcium and has been approved for the treatment of chronic hyperkalemia in chronic kidney disease (19), but studies are ongoing to assess the drug interactions between patiromer and anti-rejection medications and to assess its efficacy in the transplant recipients (20). Renal replacement therapy is reserved for severe hyperkalemia and to patients with delayed graft function or allograft failure.

## Metabolic acidosis

### Epidemiology

Metabolic acidosis after kidney transplantation is not an uncommon finding. It has been reported with varying prevalence from 12 to 58% (21). While in patients with chronic kidney disease, it is seen mainly at glomerular filtration rates (GFR) of less than 30 mL/min, in renal transplant recipients it is seen at higher GFR and even in patients with normal renal function. Factors such as suboptimal allograft function, donor age, deceased donor transplantation, graft rejection, hyperparathyroidism, and the use of calcineurin inhibitors have been associated with post-transplant acidosis (21–23). One year post kidney transplantation, the prevalence of metabolic acidosis falls to around 13–16% (21, 24).

### Pathophysiology

When present, metabolic acidosis is predominantly of the normal anion gap variant. In addition to the common pathogenic factors acting in any form of chronic kidney disease, there are other mechanisms specific to the kidney transplant status. Both proximal and distal (including type 4) renal tubular acidosis can be seen in kidney transplant recipients (25). Diarrhea causing bicarbonate loss from the gut is also a common cause of normal anion gap acidosis in post-transplant patients. Diarrhea in the post-transplant setting can also constitute a side effect of medications like mycophenolate, from known underlying bowel disease or enteric pathogens like Clostridium difficile, Cytomegalovirus infections, parasites etc.

**Figure 2:**
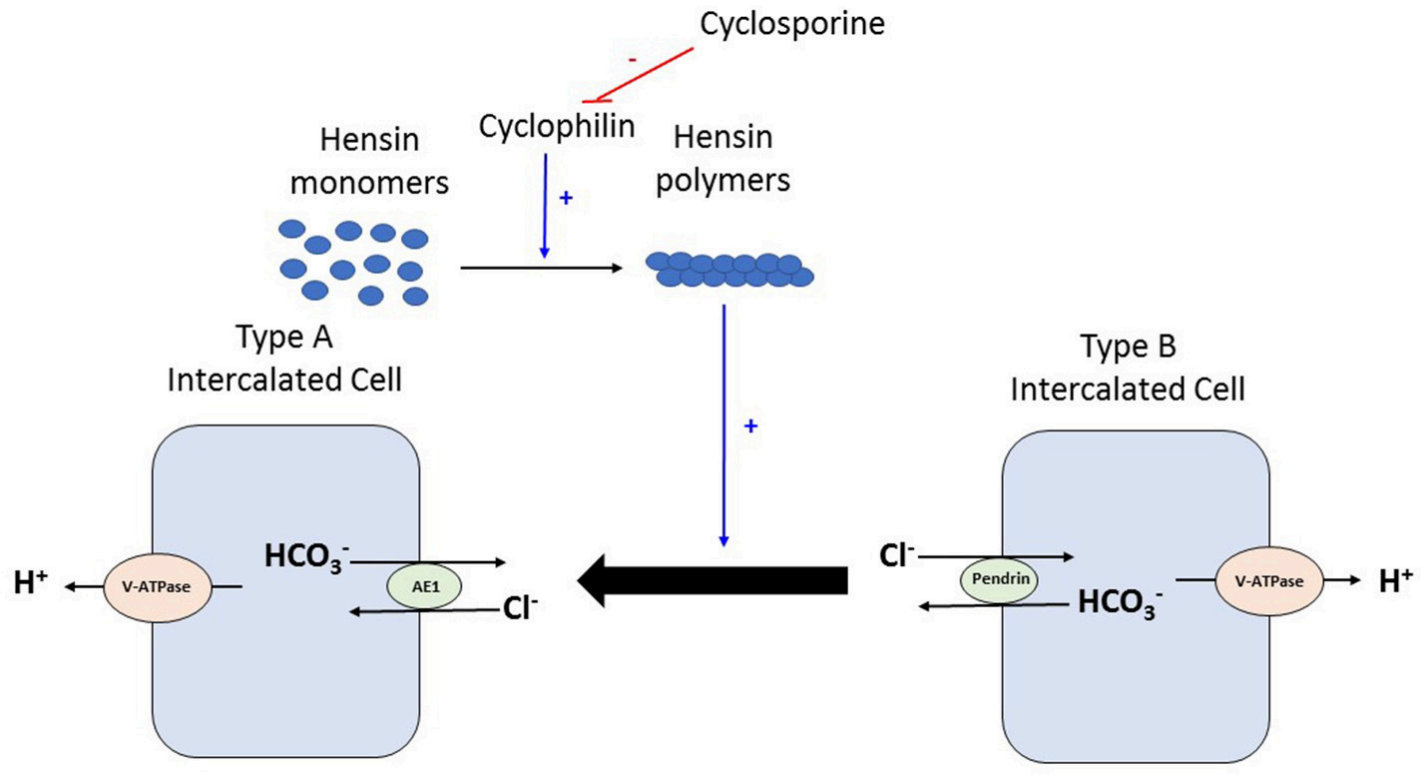
During times of acid load, type B intercalated cell converts to type Aintercalated cell to facilitate proton excretion. This process requires deposition of polymers of a protein called Hensin in the extracellular matrix. Cyclophilins are enzymes that assist in protein folding/oligomerization and are needed for polymerisation and deposition of Hensin. Cyclosporine by binding to and inhibiting the enzymatic activity of cyclophilin, prevents this adaptation of the intercalated cell from the bicarbonate secreting beta form to the acid secreting alpha form. V-ATPase, vacuolar type proton ATPase; AE, Anion exchanger 1.

Various mechanisms have been suggested for high prevalence of renal tubular acidosis in the kidney transplant recipient, which include immunological injury from rejection, subclinical tubular dysfunction from ischemia, reduced nephron mass from the single kidney processing the acid load and effect of various medications (CNIs, TMP/SMX, etc.) (26, 27). Tubulitis seen in rejection, the reduced activity of H^+^-V-ATPase pumps and anion exchanger (AE1) and decrease in renal function are postulated to be the factors responsible for association between rejection and metabolic acidosis (28). Experimental data suggests that calcineurin inhibitors impair mineralocorticoid transcriptional activity in the distal tubular cells and can thus cause aldosterone resistance contributing to hyperkalemia and metabolic acidosis in the post-transplant setting (12). Cyclosporine and tacrolimus can produce metabolic acidosis but by different mechanisms also. In the cortical collecting duct, the intercalated cell exists as a beta form that secretes bicarbonate in exchange for chloride and the alpha intercalated cell that secretes acid. Work by Watanabe et al suggests that cyclosporine produces distal renal tubular acidosis by blocking peptidyl prolyl cis-trans isomerase activity of cyclophilin and thus inhibiting this remodeling of the intercalated cell from the bicarbonate secreting beta form to the acid secreting alpha form, especially in times of acid loading (29) (Figure 2). Animal studies by Mohebbi et al. demonstrated that tacrolimus can impair urinary acidification especially in the setting of acid load by dysregulation of many acid-base transporters in the proximal and distal nephron including H^+^-ATPase, sodium bicarbonate cotransporter and anion exchanger AE1 (30). TMP/SMX can cause type 4 renal tubular acidosis often seen in association with hyperkalemia. Kidney transplantation, unrelated to immunosuppressive therapy or transplant related histologic changes, also has been shown to cause a generalized decrease in H^+^-ATPase expression and hence impaired proton handling (31).

### Clinical manifestations

Metabolic acidosis in the non-transplant population affects growth in children, causes progression of nephrocalcinosis, bone disease and is an indicator of poor outcome in chronic kidney disease at all ages (25). In the past, post-transplant renal tubular acidosis was considered asymptomatic and subclinical; however, various studies have now implicated it as a factor in bone disease, mineral metabolism and now consider it as a marker of poor renal outcome in the renal transplant recipient (22, 23, 26, 32). In a recently published retrospective cohort study of 2318 adult kidney transplant recipients, a strong detrimental association between low bicarbonate levels (less than 22 mEq/L) and graft function as well as death censored graft failure was noted even after adjusting for estimated GFR (26).

### Treatment

Starke et al performed a one-year study to assess the effect of improving bicarbonate levels on bone health in 30 kidney transplant recipients. Nineteen patients treated with potassium citrate were able to achieve bicarbonate levels of greater than 24 mEq/L and had better bone histology and markers of bone turnover when compared to 11 patients in the control group treated with potassium chloride. However, no relevant changes were seen in the DEXA scans at the end of 1 year and the study was limited by the small number of patients. In addition, this study did not comment on graft function/graft survival. Currently, given that alkali therapy is inexpensive and relatively safe, bicarbonate supplementation is recommended at least to protect the bone, if the condition is prolonged (33). It is unknown whether this supplementation will have a positive effect on renal outcomes in this population. Attention should also be paid to dietary factors, as diet might have an influence on the post-transplant acidosis. Modification of diet by higher intake of fruits and vegetables and lower animal protein intake can improve the acidosis by a small extent even in transplant recipients (34).

## Hypercalcemia

### Epidemiology

Hypercalcemia after kidney transplantation has been reported to occur with a very high variability from around 10 to 59% (35). The incidence is highest within 3 months after transplant and in majority of the patients, it resolves by 1 year after transplantation. However, in 5–10% of the patients, it persists beyond 1 year (36–38). Pre-transplant parathyroid function and longer duration of dialysis, i.e., the indicators of pre-transplant bone status, are considered risk factors for post-transplant hypercalcemia.

### Pathophysiology

Calcium is the fifth abundant mineral in our body with greater 99% being stored in the bone. Serum calcium is tightly regulated by parathyroid hormone (PTH) and 1,25-dihydroxyvitamin D3 by acting on receptors in the gut, kidney, and bone. Calcium is essential for cellular signaling, nerve impulse transmission, coagulation, muscle contraction, and bone mineralization (39). The distal nephron is responsible only for the reabsorption of 5–10% of the filtered calcium load. Yet, the distal convoluted tubule is the main regulator of calcium excretion as calcium absorption here is regulated independent of sodium absorption and is also the principal site of action of PTH, calcitonin, and vitamin D (40, 41).

Tertiary hyperparathyroidism is a very common cause of post-transplant hypercalcemia. A successful kidney transplant eliminates the stimuli that usually induce parathyroid overactivity in dialysis patients. Yet parathyroid involution does not always occur immediately, especially in patients with severe pre-transplant hyperparathyroidism, probably due to the presence of underlying parathyroid hyperplasia or adenoma, causing persistent and sometimes significant hypercalcemia. The improvement in production of calcitriol post-transplant, further stimulated by the inappropriately high PTH levels and low phosphorous levels, also is considered an important factor for the development of hypercalcemia. Steroid therapy and the resorption of soft-tissue calcium phosphate deposition formed in dialysis patients are also hypothesized as potential contributors for post-transplant hypercalcemia (35, 42–44) (see Table 1).

**Table 1:** Contributors to Hypercalcemia in kidney transplant recipients.

1. Improvement in production of calcitriol post-transplant 2. Resorption of soft-tissue calcium phosphate deposition formed in dialysis patients 3. Steroid therapy 4. Lack of parathyroid involution 5. Underlying parathyroid adenoma

### Clinical manifestations

The clinical manifestations of hypercalcemia range from anorexia, nausea, constipation, polyuria, polydipsia, and nephrocalcinosis in mild cases to cognitive difficulties, drowsiness or obtundation in severe cases in the general population (15). Given that post-transplant hypercalcemia is usually mild to moderate and is chronic in nature, such drastic symptoms are not very common. There are conflicting results in the literature about the effect of persistent hypercalcemia on graft function. Some studies consider hypercalcemia as an innocent complication that is well tolerated (45), while others have shown association with impaired graft function (46, 47), fractures, bone disease and vascular calcification.

### Treatment

KDIGO guidelines recommend that we apply the same principles for managing the mineral bone disorders in patients with chronic kidney disease stages 3–5 to managing bone mineral disorders after renal transplant. However, no specific guidelines are offered by KDIGO pertaining to the management of metabolic derangements in kidney transplant recipients with normal renal function. The first treatment option nowadays for post-transplant hypercalcemia is a calcimimetic, cinacalcet being most commonly used. Calcimimetics induce changes in conformation of the calcium receptor and thus increase sensitivity to extracellular calcium and cause reduction in PTH secretion and thereby decrease calcium levels in tertiary hyperparathyroidism (48). If there are no symptoms and patient does not have significant hypercalcemia, i.e., serum calcium greater than 11 mg/dL, most practitioners wait for at least a year for spontaneous resolution before opting for parathyroidectomy (49). Gastrointestinal side effects and cost of cinacalcet can limit the use of cinacalcet in some patients. Parathyroidectomy is more cost effective when cinacalcet duration reaches 14–16 months (50, 51).

Evenepoel et al. studied the effect of cinacalcet in a placebo-controlled trial for management of post-transplant hypercalcemia due to persistent hyperparathyroidism. They demonstrated that cinacalcet normalizes serum calcium and lowers PTH levels while placebo treatment led to no significant changes in these parameters and the discontinuation rate was quite low. However, after 1 year of treatment, the cinacalcet group had several other outcomes similar to placebo treatment: no improvement in bone mineral density (BMD), no improvement in biomarkers of high bone turnover and both groups had comparable and stable estimated GFR (52). When they looked at serum calcium and PTH levels after withdrawal of cinacalcet, the levels were similar in the cinacalcet and placebo groups, suggesting that cinacalcet did not hasten parathyroid gland involution. The follow up period in this study was relatively short; however, this study underlines the dilemma that continues when prescribing calcimimetics. There is definite improvement in PTH and calcium levels while using cinacalcet, but the studies that have shown improvement in hard outcomes like BMD are few and some studies in turn indicate a concern for low bone turnover state and decrease in BMD (53–55). Cruzado et al randomized 30 post-transplant patients with hyperparathyroid hypercalcemia to receive cinacalcet or subtotal parathyroidectomy. The surgery group in the study had greater reduction of parathyroid hormone levels and was associated with a significant increase in femoral neck BMD but also had higher incidence of hypocalcemia. No significant change in vascular calcification was seen in either group (50). Future large randomized trials that assess hard clinical outcomes such as bone disease and graft function would help guide the management of hypercalcemia in the post-transplant population.

## Hypomagnesemia

### Epidemiology

In renal transplant recipients, hypomagnesemia is reported with high prevalence with lowest serum magnesium concentration noticed around second month post transplantation. In 20% of the renal transplant recipients, hypomagnesemia might persist several years after transplantation (56). The incidence of hypomagnesemia and that of post-transplant diabetes (previously referred as NODAT) is reported to be higher among patients using tacrolimus than those on cyclosporine (57–59).

### Pathophysiology

Magnesium is the fourth most abundant cation in the body and the second most abundant intracellular cation after potassium (60). Around 99% of body magnesium is found in bone, muscle and soft tissues. Renal excretion is the major regulator of magnesium balance of the body and plays a crucial role in magnesium homeostasis. Bulk of the reabsorption of the filtered magnesium occurs in the thick ascending loop of Henle where magnesium is reabsorbed via the paracellular pathway facilitated by tight-junction proteins Claudin 16 and 19. Magnesium reabsorption in this segment depends on lumen-positive voltage created by potassium recycling in the thick ascending limb cells. Around 10% of the filtered magnesium is reabsorbed in the DCT via TRPM6 (transient receptor of potential melastatin 6) transporters, playing an important role in determining the final urinary magnesium concentration (61).

**Figure 3:**
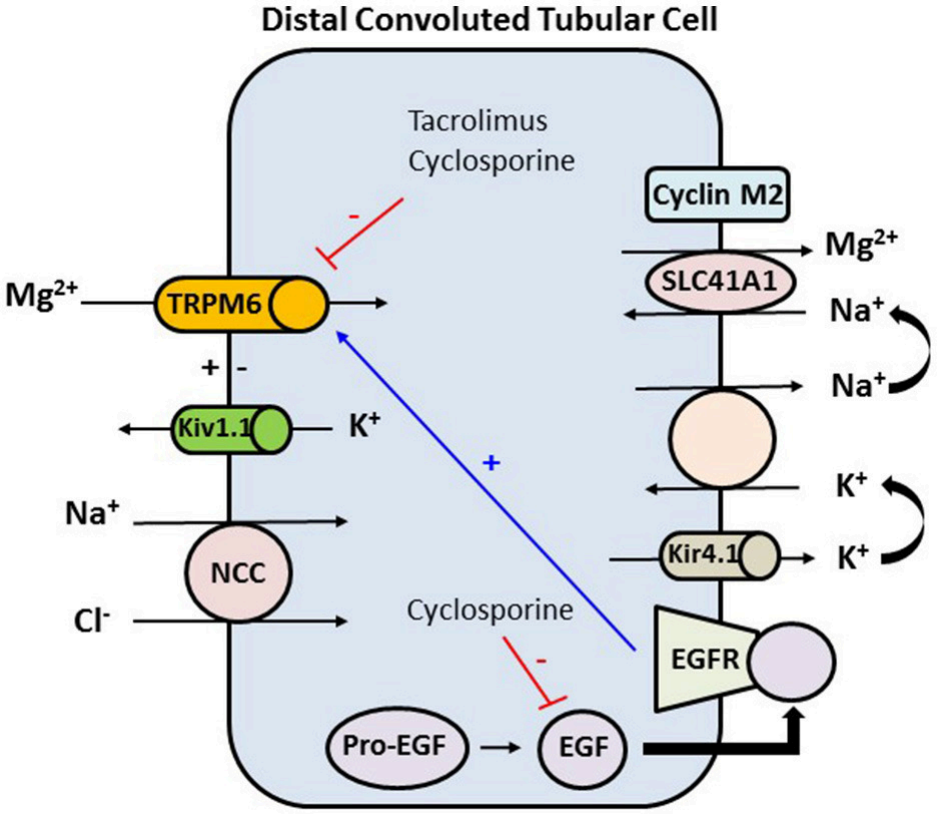
In the distal convoluted tubule the final 10% of magnesium is reabsorbed in an active transcellular manner. The tubular epithelium in this part has a lumen-negative voltage. Apical reabsorption occurs via TRPM6, which can be stimulated via the basolateral EGFR. Kv1.1, an apically located potassium channel, establishes a favorable luminal membrane potential facilitating an increase in the driving force for magnesium reabsorption via TRPM6. At the basolateral membrane, magnesium is extruded via an unknown mechanism likely SLC41A1 transporter, which may be regulated by cyclin M2 acting as magnesium sensor. Magnesium extrusion depends on the sodium gradient, set by the Na+-K+-ATPase. The activity of the Na+-K+-ATPase is in turn dependent on potassium recycling via Kir4.1 channel. Tacrolimus and cyclosporine downregulate TRPM6 which is the major active transport protein in the distal tubule that is needed for reabsorption of magnesium. Cyclosporine also decreases the expression of EGF, and hence decreasing the expression of TRPM6. TRPM6, transient receptor potential melastatin type 6; Kv1.1, apical voltage-gated K channel 1.1; NCC, sodium chloride cotransporter; SLC41A1, solute carrier family 41 A1 Mg2+ transporter; Kir4.1, basolateral voltage-gated channel; EGF, epidermal growth factor; EGFR, epidermal growth factor receptor.

Use of CNIs is associated with an inappropriate and significant increase in fractional excretion of calcium and magnesium. CNIs induce renal magnesium wasting by downregulation of magnesium transport proteins in the distal tubule (Figure 3). CNIs induce decrease in expression of the magnesium transporter TRPM6 in the distal tubule, which is the main site for active transcellular reabsorption for magnesium (62). This decrease in expression of magnesium transport proteins leads to increased magnesium excretion, causing persistently elevated fractional excretion of magnesium despite hypomagnesemia in these patients. Some recent data suggest that cyclosporine downregulates renal epidermal growth factor (EGF) production, which in turn leads to inhibition of TRPM6 activation (63, 64). This magnesium wasting effect is seen even with use of low dose of CNIs (64). Use of steroids, hyperglycemia, diuretics, and proton pump inhibitors could be the other contributing factors to hypomagnesemia in the early post-transplant period.

### Clinical manifestations

Magnesium is an essential cofactor for critical enzymatic reactions, and hence has a role in various physiological functions involving the neuromuscular and cardiovascular systems. Magnesium plays a role in PTH-induced release of calcium from bone and in severe hypomagnesemia also might cause diminished PTH secretion (65). Hypomagnesemia can cause tremors, tetany, and convulsions and in some cases hypokalemia. Initial EKG changes in hypomagnesemia manifest as widening of the QRS complex and peaking of T waves. Severe cases can lead to prolonged PR interval, progressive widening of the QRS complex, and diminution of the T wave (66). Even mild hypomagnesemia has been associated with ventricular arrhythmias in patients with underlying cardiac disease.

There are studies showing association of hypomagnesemia with vascular stiffness and decreased graft survival in patients with CNI toxicity (67–70). Gupta et al. looked at 14 hypomagnesemic nondiabetic renal transplant recipients and their effect on lipid and glucose metabolism over a 6-month period. This was a small and short term study, however their results suggest that correction of hypomagnesemia in renal transplant recipients was associated with reduced serum total cholesterol, LDL, and total cholesterol/HDL ratio (71).

It has been well established that there is a strong association between hypomagnesemia and insulin resistance in the general population. Due to increasing awareness about post-transplant diabetes, several studies looked at hypomagnesemia after renal transplantation and its relation with post-transplant diabetes. Intracellular Magnesium regulates enzymes and ion transport channels in pancreatic beta cells and plays a role in insulin receptor phosphorylation. Conversely, insulin activates the renal magnesium channel TRMP6 that plays a role in magnesium reabsorption in the kidney and hence plays a role in magnesium homeostasis. Consequently, it is suggested that a vicious cycle may ensue in hypomagnesemic patients with diabetes in which low magnesium levels causes insulin resistance and insulin resistance reduces serum magnesium concentrations (72).

Huang et al. from University of Toronto looked at 948 nondiabetic kidney transplant recipients to examine the relationship between serum magnesium level and post-transplant diabetes during a median follow up of 3.4 years. Lower plasma magnesium level (defined as a plasma magnesium less than 1.8 mg/dL) in their study was associated with a quantitatively increased risk of post-transplant diabetes, based on time-fixed, conventional time-varying, and rolling-average time-varying Cox proportional hazards models. (Hazard ratio of 1.58–1.83, *p* < 0.05) (73).

### Treatment

Though several such studies exist that show an independent association between hypomagnesemia and post-transplant diabetes (74, 75), treating it by way of magnesium supplementation has not always translated into beneficial results in clinical studies. Van Laecke et al. conducted an open-label study to assess if magnesium supplementation improved glycemic control in renal transplant recipients. They randomized 54 patients within 2 weeks after kidney transplantation to receive magnesium oxide supplementation (with goal serum magnesium greater than 1.9 mg/dl) or no treatment. Patients on magnesium supplementation displayed lower fasting plasma glucose at 3 months post-transplant compared with controls. However, the effect was not large (104.1 mg/dl in the control group vs. 92.6 mg/dl in the treatment group, *p* = 0.02), and study had the drawback that fasting plasma glucose levels were higher in the control group already at baseline. In addition, no significant differences were noted in other measures of glycemia i.e., area under the glucose curve during an oral glucose tolerance test and in insulin resistance as expressed by Homeostasis Model of Assessment-Insulin Resistance (HOMA-IR). The trial was also underpowered to evaluate the effect of magnesium supplementation on the risk of post-transplant diabetes. Of note, one in four patients in the treatment group had persistent hypomagnesemia despite reasonably high doses of magnesium oxide (76).

There are conflicting reports about magnesium levels adequately improving with magnesium supplementation in patients on CNIs due to high rate of renal excretion with CNIs. While some studies demonstrated that serum magnesium levels increased to normal range after magnesium oxide therapy (71, 77), other studies did not demonstrate an adequate improvement in levels despite supplementation (57, 74). While magnesium supplementation for patients with type 2 diabetes mellitus has shown improvement in glucose metabolism and insulin sensitivity, the same results have not been demonstrated in renal transplant recipients so far. Magnesium therapy is usually well tolerated but gastrointestinal side effects are not infrequent.

As there are controversies yet to be answered whether magnesium supplementation would help any of the above outcomes, it is reasonable to supplement magnesium in all patients with even mild hypomagnesemia if underlying cardiovascular disease is present. Table 2 list commonly used magnesium salt preparations for oral supplementation in kidney transplant recipients. In post renal transplant patients with mild hypomagnesemia and no risk factors, it is reasonable to at least advise foods rich in magnesium and reserve supplementation for moderate (less than 1.5 mg/dl) and severe hypomagnesemia (less than 1.2 mg/dl). Intravenous magnesium supplementation followed by oral supplementation should be reserved for severe hypomagnesemia and in symptomatic patients. The population in the United States does not usually meet the expected average requirement for magnesium from food consumed due to change in dietary habits and move toward more processed foods in recent years. In plants, magnesium forms the central ion of chlorophyll and hence magnesium is abundant in green leafy vegetables. It is also found in abundance in other foods such as legumes, nuts, brown rice, unprocessed cereals such as whole grains, and whole wheat bread (60, 78).

**Table 2:** Examples of Magnesium preparations available in USA.

**Type of preparation**	**Packager**	**Form and strength of salt**	**Elemental magnesium content in dosage form**	Dosage form^*^	**GI absorption^†^**
Magnesium Oxide^1^	Generic pharmaceutical	400 mg tablet	241.3 mg	19.86 mEq	15–30%
Magnesium L-lactate dihydrate^2^	Brandywine pharmaceuticals, Inc.	84 mg tablet	84 mg	7 mEq	40–60%
Magnesium hydroxide^3^	Amerisource Bergen	1,200 mg per 15 ml liquid	39.6 mg/ml	3.3 mEq/ml	Poorly bioavailable. Not used as magnesium supplement.
Magnesium chloride^4^	Mylan Institutional LLC	200 mg/ml injection	24 mg/ml	1.97 mEq/ml	N/A
Magnesium Sulfate Heptahydrate^5^	Fresenius Kabi USA, LLC	500 mg/mL	49.3 mg/ml	194.7 mEq/ml	N/A

* Values are approximate.

† Absorption in oral preparations is variable-depends on dietary factors, gastric pH and has interpatient variability. N/A = Not applicable.

1 DailyMed. MAGNESIUM OXIDE- Magnesium Oxide Tablet. Available online at: https://dailymed.nlm.nih.gov/dailymed/drugInfo.cfm?setid=9e35187e-0d1d-40cd-8668-65b221243908 (Accessed January 11, 2017).

2 *DailyMed. MAGNESIUM L-LACTATE DIHYDRATE. Available online at: https://dailymed.nlm.nih.gov/dailymed/drugInfo.cfm?setid=65f20da4-9183-47df-bdf1-9b6f2d964f20 (Accessed April 10, 2018)*.

3 *DailyMed. GOOD NEIGHBOR PHARMACY MILK OF MAGNESIA- Magnesium Hydroxide Suspension. (Accessed May 6, 2016)*.

4 DailyMed. MAGNESIUM CHLORIDE- Magnesium Chloride Injection. Available online at: https://dailymed.nlm.nih.gov/dailymed/drugInfo.cfm?setid=2105d1c1-54ef-4d1a-a63c-b1619e8b50db. (Accessed March 14, 2013).

5 *DailyMed. MAGNESIUM SULFATE- Magnesium Sulfate Heptahydrate Injection, Solution. (Accessed April 3, 2014)*.

## Hypophosphatemia

### Epidemiology

Hypophosphatemia in kidney transplant recipients has been reported with varying incidence from 40 to 93% in different studies (45, 79–81). Data indicate that the incidence of hypophosphatemia peaks at week two post-kidney transplant and renal phosphate wasting usually regresses by 1 year after successful kidney transplantation (82, 83).

### Pathophysiology

Phosphate is a major player in energy metabolism, bone formation, nucleic acid, and cell membrane formation and in several signaling pathways. Majority of total body phosphate is stored in the bone, with less than one percent present in the extracellular fluid. Maintaining this small percentage of total body phosphate in the narrow range of 2.5 to 4.5 mg/dl in the serum is mainly achieved by the kidneys via regulation of renal phosphate reabsorption in the proximal tubule. The renal sodium phosphate cotransporters NaPiIIa and NaPiIIc in the proximal tubule use the energy derived from the transport of sodium down its gradient to move inorganic phosphate from the lumen into the cell and are responsible for reabsorbing 85% of the filtered phosphate in the tubule (41).

Dietary phosphate, parathyroid hormone, vitamin D, insulin and fibroblast growth factor 23 (FGF23) are the major regulators of phosphate homeostasis and they involve a complex interplay between intestinal absorption, internal redistribution, and renal excretion of phosphate. FGF23 is a recently characterized phosphaturic hormone that is produced in osteoblasts in response to increased phosphate levels. FGF23 causes suppression of vitamin D 1α hydroxylation, increase in renal phosphate excretion, and decreases gut absorption of phosphate causing a decrease in serum phosphate levels. Vitamin D increases phosphate absorption in gut and kidney while PTH increases renal excretion of phosphate (84).

FGF23 and PTH are found to be at elevated levels in chronic kidney disease and end stage renal disease patients in response to elevated phosphate levels. After successful kidney transplantation, elevated FGF23 levels and other phosphatonins might take some time to come down and this persistent elevation is considered to play a key causative role in the phosphaturia and hypophosphatemia seen in the kidney transplant recipients (85, 86). Tacrolimus also increases renal phosphate wasting by decreasing the abundance of phosphate cotransporter NaPi-2a in the renal tubule (30, 87). Factors such as dialysis vintage, low calcitriol levels, elevated parathyroid hormone level, and tubular damage from immunosuppression are thought to be the other contributors that determine phosphate wasting after renal transplantation (81, 86, 88, 89).

### Clinical manifestations

Hypophosphatemia, especially in the severe forms is known to cause predisposition to hemolysis, rhabdomyolysis, respiratory failure, muscle weakness, seizures, and myocardial depression (15). There is growing concern that hypophosphatemia can cause muscle weakness, osteodystrophy and contribute to increased fracture risk in this patient population. However, no association exists between renal phosphate wasting and adverse graft outcomes (45, 90). In fact, some newer studies actually show favorable outcomes in patients with hypophosphatemia likely indicating a very-well functioning graft (91).

### Treatment

Though no specific guidelines exist for the management of post-transplant hypophosphatemia, patients are usually treated with oral phosphate supplements and the treatment is well tolerated. However, intervention studies are scarce for the treatment of post-transplant hypophosphatemia and phosphate supplementation in these patients is not always considered benign. There is a theoretical risk that phosphate supplementation can in turn increase PTH and FGF23 synthesis and hence worsen hyperparathyroidism and cause further phosphate wasting in patients with a well-functioning allograft (44, 92). This effect was not seen in the early period after kidney transplantation, but was seen in late post-transplant periods in some studies. (80, 93). Patients with persistent post-transplant hyperparathyroidism with concurrent hypercalcemia and hypophosphatemia can be treated effectively with cinacalcet (48, 52). Kidney transplant recipients should be advised to consume phosphate rich foods like whole grains, eggs, poultry, fish, and dairy products as soon as good graft function is achieved (94, 95).

## Conclusion

There is high incidence and prevalence of a spectrum of acid–base and electrolyte disorders in renal transplant recipients. Understanding the pathophysiological effects of these disorders and knowledge of the current available evidence regarding therapy is crucial for making effective clinical decisions in this vulnerable population. Additional research in both fundamental and clinical domains is necessary to ensure an evidence-based approach for the management of these disorders.

## Author contributions

VP has written the sections of hyperkalemia, metabolic acidosis, hypocalcemia, hypomagnesemia, and hypophosphatemia with some contribution of HR-B. VP and HR-B contributed equally to developing the tables, introduction, and conclusions. HR-B developed all the figures.

### Conflict of interest statement

The authors declare that the research was conducted in the absence of any commercial or financial relationships that could be construed as a potential conflict of interest.
